# Increased Numbers of NK Cells, NKT-Like Cells, and NK Inhibitory Receptors in Peripheral Blood of Patients with Chronic Obstructive Pulmonary Disease

**DOI:** 10.1155/2013/721782

**Published:** 2013-08-31

**Authors:** Ying Tang, Xiaodan Li, Man Wang, Qi Zou, Shasha Zhao, Bowen Sun, Lijun Xu, Yanfang Jiang

**Affiliations:** ^1^Department of Respiratory Medicine, The First Hospital, Jilin University, Changchun 130021, China; ^2^Department of the Central Laboratory, The Second Part of the First Hospital, Jilin University, Changchun 130021, China

## Abstract

T cells and B cells participate in the pathogenesis of COPD. Currently, NK cells and NKT cells have gained increasing attention. In the present study, 19 COPD patients and 12 healthy nonsmokers (HNS) were recruited, and their pulmonary function was assessed. The frequencies of CD3^+^ T, CD4^+^ T, CD8^+^ T, B, NK, and NKT-like cells were determined using flow cytometry. The frequencies of spontaneous and inducible IFN-**γ** ^+^ or CD107a^+^ NK and NKT-like cells as well as activating or inhibitory receptors were also detected. The potential association of lymphocyte subsets with disease severity was further analyzed. Significantly decreased numbers of CD3^+^ and CD4^+^ T cells, and the CD4^+^/CD8^+^ ratio, but increased numbers of CD3^−^CD56^+^ NK and CD3^+^CD56^+^ NKT-like cells were observed in COPD patients compared to HNS. The frequencies of inducible IFN-**γ**-secreting NK and NKT-like cells were less in COPD patients. The frequencies of CD158a and CD158b on NK cells and CD158b on NKT-like cells were greater. The frequency of CD158b^+^ NK cells was negatively correlated with FEV_1_% prediction and FEV_1_/FVC. Our data indicate that COPD patients have immune dysfunction, and higher frequencies of inhibitory NK cells and NKT-like cells may participate in the pathogenesis of COPD.

## 1. Introduction

Chronic obstructive pulmonary disease (COPD) is one of the most prevalent chronic adult diseases, affecting more than 200 million people worldwide, and has become the fourth leading cause of death [1]. The term includes a group of pulmonary disorders that cause dyspnea, which are characterized by structural changes in the lung and lead to progressive irreversible airflow limitation [2]. COPD is frequently associated with immune dysregulation, which makes the patients prone to infections. The number of CD4^+^ T lymphocytes and the CD4^+^/CD8^+^ ratio have been shown to be less in COPD patients compared with healthy volunteers [3–5], while the numbers of CD8^+^ T cells and B cells increase as COPD progresses [6–8].

In recent years, natural killer (NK) cells and natural killer T (NKT) cells have gained increasing attention. NK cells and NKT cells are the first line of defense against infection in the innate immune system, and they have the ability to directly kill target cells and interact with antigen-presenting cells as well as T cells [9–12]. NKT cells are innate-like T cells that constitute a minor lymphocyte population and exhibit features of both T cells and NK cells. Upon stimulation, NKT cells rapidly produce large amounts of cytokines [13]. Prieto et al. have revealed a functional defect in NK cells of patients with COPD; however, the number of NK cells does not differ significantly in peripheral blood between COPD patients and healthy controls [14]. Urbanowicz et al. have shown reduced NK cells and NKT cells in terms of both numbers and cytotoxicity in peripheral blood, while there were not any differences in cytokine production in these cells [15]. Currently, the numbers and function of NK cells in COPD patients are controversial, and few in-depth studies have focused on the role of NKT cells in COPD.

NK cell function is determined by the integration of signals arising from the engagement of different NK receptors with specific ligands on potential target cells [16]. NK cell receptors can be divided into activating receptors (such as NKG2C, NKG2D, NKp30, and NKp46) and inhibitory receptors (such as CD158a, CD158b, KIR3DL1, and NKG2A) [17]. The presence of these receptors is not exclusive to NK cells, as NKT cells also express these similar receptors [18]. The inhibitory receptors discriminate healthy from diseased cells by surveying the surface expression of self-MHC class I molecules and prevent the attack of NK cells against these cells [19–22]. The activating receptors bind to host-derived or pathogen-encoded ligands, which are upregulated on stressed or infected cells. Once activated, NK cells and NKT cells can produce inflammatory cytokines, such as IFN-*γ*, and directly lyse target cells by exocytosis of perforin and granzyme [23]. However, little is known about NK cell and NKT cell receptors in COPD.

In order to verify the role of NK cells and NKT cells in COPD patients, we detected the numbers of CD3^−^CD56^+^ NK cells and CD3^+^CD56^+^ NKT-like cells from the peripheral blood of COPD patients and compared their values with those in healthy nonsmokers (HNS) who acted as controls. We found that the numbers of CD3^−^CD56^+^ NK cells and CD3^+^CD56^+^ NKT-like cells were greater, while the functions were impaired in COPD patients. The numbers of CD158a^+^ and CD158b^+^ NK cells and CD158b^+^ NKT-like cells were greater in COPD patients, and the frequency of CD158b^+^ NK cells was negatively correlated with the forced expiratory volume in one second (FEV_1_)% prediction and the ratio of FEV_1_ to forced vital capacity (FVC).

## 2. Materials and Methods

### 2.1. Patients and Controls

Nineteen COPD patients were recruited from the outpatient clinic of the First Hospital of Jilin University from October 2011 to October 2012. Twelve HNSs from the medical examination center were recruited as controls. The patients were diagnosed as having COPD if their FEV_1_/FVC ratio was less than 70% and the FEV_1_ was predicted to be less than 80% after inhaling a bronchodilator. All 19 COPD patients inhaled salmeterol (50 *μ*g twice daily) plus fluticasone propionate (500 *μ*g twice daily) for long-term therapy. Participants were excluded if they had a history of tuberculosis, a clinical suspicion of current infection, or a history of physician-diagnosed asthma. COPD subjects were also excluded if they had an exacerbation within the previous 6 weeks, other pulmonary diseases, or severe functional defects of the heart, liver, or kidney. The patient characteristics are summarized in Table 1, and the lymphocyte subsets of the two groups were compared. The study was approved by the Human Ethics Committee of Jilin University, ChangChun, China. Written informed consent was obtained from each participant.

### 2.2. Pulmonary Function

Spirometry was performed using a dry rolling-seal spirometer (JAEGER Master Screen, USA). A standardized spirometry protocol, which exceeded the American Thoracic Society (ATS) testing standards, and a strict quality control program were used to obtain acceptable and reproducible data [24]. Values obtained were FEV_1_% prediction and the FEV_1_/FVC ratio. The FEV_1_% prediction and FEV_1_/FVC ratio should be more than 80% and 70% of the prediction, respectively, in normal pulmonary function.

### 2.3. Computed Tomography (CT) Scan

CT is more sensitive than plain radiographs, and it allows for a more detailed evaluation of the pulmonary parenchyma and surrounding structures. All patients underwent a CT scan. The COPD patients showed hyperinflation and enhancement of pulmonary markings.

### 2.4. Flow Cytometry of Lymphocyte Subsets

Human peripheral blood samples were stained with various monoclonal antibodies (mAbs) to determine the frequency of lymphocyte subsets in each individual using flow cytometry. The following mAbs and reagents were used in this study: fluorescein-isothiocyanate- (FITC-) conjugated anti-CD4 mAb, phycoerythrin- (PE-) conjugated anti-CD8 mAb, peridinin-chlorophyll-protein- (PerCP-) conjugated anti-CD3 mAb (Clone SK7/SK1/SK3, BD Tritest, San Jose, CA, USA), FITC-conjugated anti-CD3 mAb, and phycoerythrin- (PE-) conjugated anti-CD19 mAb (Clone SK7/4G7, BD Tritest, San Jose, CA, USA). Briefly, 100 *μ*L of blood was used for each staining experiment, and the samples were incubated for 30 min at room temperature. Erythrocytes were lysed using BD FACS lysing Solution 2 (BD Bioscience, San Diego, CA, USA). After washing with phosphate-buffered saline (PBS), the cells were subjected to flow cytometric analysis using a FACS Calibur (Beckton Dickinson) and FlowJo software (v5.7.2) (TreeStar, Ashland, OR, USA).

### 2.5. Flow Cytometry of NK Cells, NKT-Like Cells, and NK Receptors

Individual venous blood samples were subjected to flow cytometric analysis using specific antibodies. Briefly, 100 *μ*L of blood samples was incubated in a mixture of FITC-conjugated anti-CD3, allophycocyanin- (APC-) conjugated anti-CD56 (Clone B159, BD Pharmingen, San Diego, CA, USA), PerCP-conjugated anti-CD16 (Clone 3G8, BD Pharmingen, San Diego, CA, USA), and phycoerythrin- (PE-) conjugated anti-NKG2C (Clone 134591, R&D Systems Inc., Minneapolis, MN, USA), anti-NKG2D (Clone 1D11, Biolegend, San Diego, CA, USA), anti-NKp30 (Clone p30-15, BD Bioscience, San Jose, CA, USA), anti-NKp46 (Clone 9E2/NKp 46, BD Bioscience, San Jose, CA, USA), anti-NKG2A (Clone 131411, R&D Systems Inc., Minneapolis, MN, USA), anti-KIR3DL1 (Clone VL 186-1.6, Biolegend, San Diego, CA, USA), anti-CD158a (Clone HP-3E4, BD Bioscience, San Jose, CA, USA), and anti-CD158b (Clone CH-L, BD Bioscience, San Jose, CA, USA) for 30 min at room temperature, respectively. Mouse IgG1 (Clone MOPC-21, BD Pharmingen, San Diego, CA, USA) and IgG2a (Clone G155-178, BD Pharmingen, San Diego, CA, USA) were used as negative controls. The remaining erythrocytes were lysed using BD FACS lysing Solution 2 (BD Bioscience, San Jose, CA, USA), and the frequencies of different subsets of NK cells were determined by flow cytometry using a FACS Calibur (BD Bioscience) and FlowJo software (v5.7.2).

### 2.6. The Detection of IFN-*γ* Secreted by NK and NKT-Like Cells

Peripheral blood mononuclear cells (PBMCs) were isolated from individual heparinized blood samples by density-gradient centrifugation using Ficoll-Paque Plus (Amersham Biosciences, Little Chalfont, UK). Next, PBMCs (10^6^ cells/well) were incubated in complete RPMI 1640 culture medium in the presence of 50 ng/mL phorbol myristate acetate (PMA; Sigma-Aldrich, St. Louis, MO, USA) and 1.0 *μ*g/mL ionomycin (Sigma-Aldrich) for 2 h at 37°C in 5% CO_2_. Thereafter, Brefeldin A (GolgiPlug; Becton Dickinson) was added to the cultures, which were incubated for 4 h. After incubation for 6 h, PBMCs were then washed with 2 mL of cold PBS and resuspended in cytometry buffer. Surface staining with anti-CD56 (Clone B159, BD Pharmingen, San Diego, CA, USA) and anti-CD3 mAbs (Clone HIT3a, BD Pharmingen, San Diego, CA, USA) was performed as described above. For intracellular staining of IFN-*γ*, PBMCs were fixed with 100 *μ*L of 4% paraformaldehyde (30 min at room temperature) and then permeabilized with 300 *μ*L of 0.5% saponin plus 10% fetal bovine serum in PBS (30 min at room temperature). After several washes, PBMCs were stained with anti-IFN-*γ* PE (Clone 4S.B3, BD Pharmingen, San Diego, CA, USA) (30 min at room temperature). Finally, PBMCs were washed with cytometry buffer and stored at 4°C until analysis.

### 2.7. The Degranulation of NK and NKT-Like Cells

PBMCs were isolated as described above. The PBMCs (10^6^ cells/well) were cocultured in duplicate with K562 cells at a ratio of 10 : 1 of effector to target (E : T) in RPMI-1640 medium (Invitrogen, Carlsbad, CA, USA) in the presence of anti-CD107a (Clone H4A3, BD Pharmingen, San Diego, CA, USA) or control IgG2a (Clone G155-178, BD Pharmingen, San Diego, CA, USA) for 6 h. The PBMCs cultured alone served as negative controls. Subsequently, the cells (10^6^/tube) were stained in duplicate with FITC-anti-CD3 and APC-anti-CD56 at room temperature for 30 min, respectively. After washing with PBS (containing 1% fetal calf serum and 2.5% paraformaldehyde), the frequencies of CD107a^+^CD3^−^CD56^+^ NK cells and CD107a^+^CD3^+^CD56^+^ NKT-like cells were determined by flow cytometric analysis using a FACS Calibur; at least 10,000 events per sample were analyzed.

### 2.8. Statistical Analysis

Data are expressed as average ± standard deviation (SD) unless specified otherwise. The differences between the two groups were analyzed by the Wilcoxon rank sum test using SPSS 18.0 software. The relationship between two variables was evaluated using the Spearman rank correlation test. A two-sided *P* value <0.05 was considered statistically significant.

## 3. Results

### 3.1. Imbalance of Immune Function in COPD Patients

**Figure 1:**
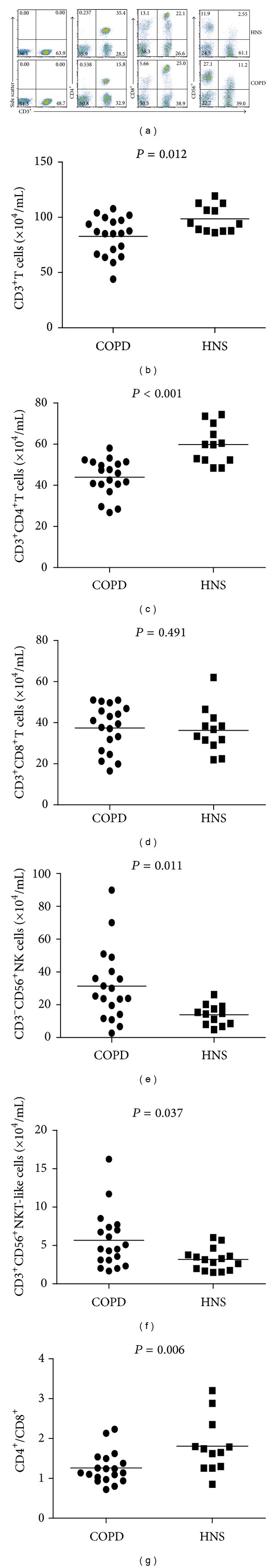
Lymphocyte subsets in COPD patients and HNS. (a) The cells were gated initially on living lymphocytes. At least 50,000 events were analyzed for each sample. Viable lymphocytes were gated on the basis of forward and side angle light scattering characteristics. Data shown are representative charts from different groups of subjects and the frequencies of CD3^+^ T cells, CD4^+^ T cells, CD8^+^ T cells, NK cells, and NKT-like cells in individual subjects. (b), (c), (d), (e), and (f) Summarized data show the number of CD3^+^ T cells, CD3^+^ CD4^+^ T cells, CD3^+^CD8^+^ T cells, CD3^−^CD56^+^ NK cells, and CD3^+^CD56^+^ NKT-like cells in COPD patients and HNS. (g) Summarized data show the CD4^+^/CD8^+^ ratio. The horizontal lines show the median.

To confirm the change of lymphocyte subsets in COPD patients, we detected the numbers of T cells, B cells, and NK cells from the peripheral blood of COPD patients by flow cytometry. Our research revealed that the numbers of CD3^−^CD56^+^ NK cells and CD3^+^CD56^+^ NKT-like cells were greater (*P* = 0.011, *P* = 0.037, resp.), while the numbers of CD3^+^ T cells, CD4^+^ T cells, and the CD4^+^/CD8^+^ ratio were less (*P* = 0.012, *P* < 0.001, *P* = 0.006, resp.) in COPD patients compared with HNS (Figures 1(b)-1(c), 1(e)–1(g)). The number of CD8^+^ T cells was not statistically different in COPD patients compared with HNS (*P* = 0.491, Figure 1(d)). In addition, there was no significant difference in the numbers of B cells and CD3^−^CD16^+^ NK cells between COPD patients and HNS (data not shown).  Figure 1(a) shows the representative charts of CD3^+^ T cells, CD4^+^ T cells, CD8^+^ T cells, CD3^−^CD56^+^ NK cells, and CD3^+^CD56^+^ NKT-like cells in individual subjects from different groups.

### 3.2. Functionally Impaired NK Cells and NKT-Like Cells in COPD Patients

**Figure 2:**
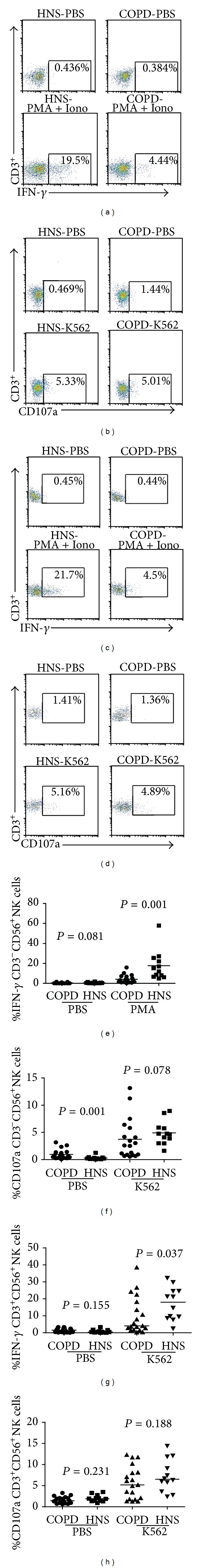
The frequencies of inducible IFN-*γ*-secreting NK cells and NKT-like cells and CD107a^+^ NK cells and NKT-like cells in COPD patients and HNS. (a) and (b) The cells were gated initially on CD3^−^CD56^+^ NK cells. At least 10,000 events were analyzed for each sample. Data shown are representative charts from different groups of subjects and the frequencies of IFN-*γ*-secreting NK cells and CD107a^+^ NK cells in individual subjects. (c) and (d) The cells were gated initially on CD3^+^CD56^+^ NK cells. Data shown are representative charts from different groups of subjects and the frequencies of IFN-*γ*-secreting NKT-like cells and CD107a^+^ NKT-like cells in individual subjects. (e) and (f) Summarized data show the frequency of IFN-*γ*-secreting NK cells and CD107a^+^ NK cells in individual subjects. (g) and (h) Summarized data show the frequency of IFN-*γ*-secreting NKT-like cells and CD107a^+^ NKT-like cells in individual subjects.

Our previous research indicated that the numbers of NK cells and NKT-like cells were greater in COPD patients compared to HNS. Subsequently, we studied the functions of NK cells and NKT-like cells by detecting the secretion of IFN-*γ* and CD107a degranulation in NK cells and NKT-like cells. As shown in Figures 2(b), 2(d), 2(f), and 2(h), there were no significant differences in the frequencies of CD107a^+^ NK cells and CD107a^+^ NKT-like cells stimulated by K562 cells between patients and controls, while the frequencies of IFN-*γ*-secreting NK cells and NKT-like cells stimulated by PMA and ionomycin were significantly less in COPD patients than in controls (*P* = 0.001, *P* = 0.037, Figures 2(a), 2(c), 2(e), and 2(g)).

### 3.3. Increased Numbers of CD158a^+^ NK Cells, CD158b^+^ NK Cells, and CD158b^+^ NKT-Like Cells in COPD Patients

**Figure 3:**
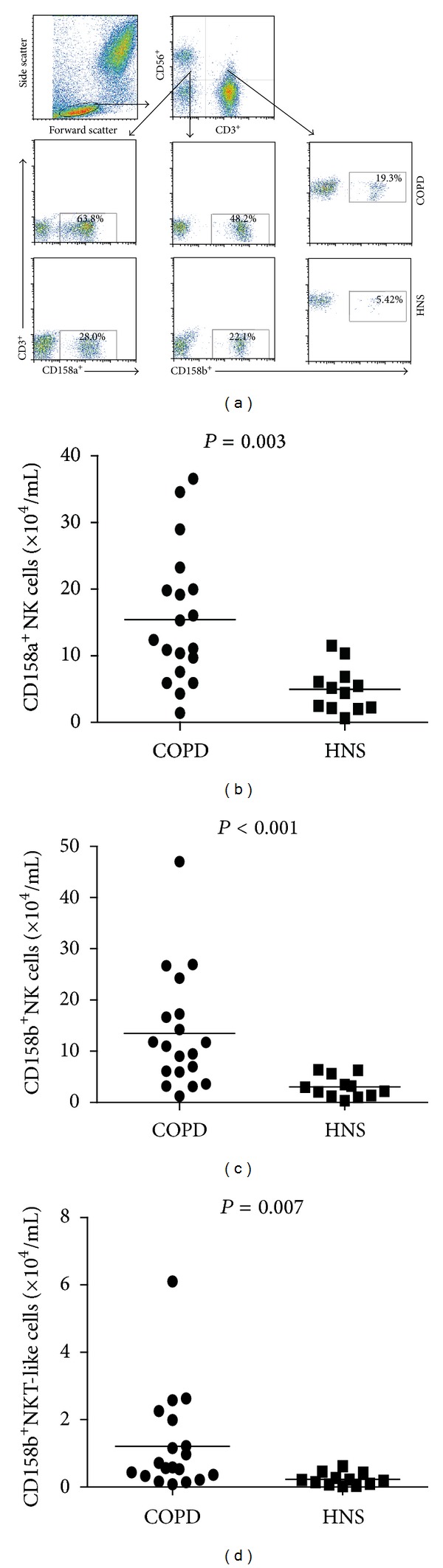
CD158a^+^ NK cells, CD158b^+^ NK cells, and CD158b^+^ NKT-like cells in COPD patients. (a) Data shown are representative charts from different groups of subjects and the frequencies of CD158a^+^, CD158b^+^ NK cells, and CD158b^+^ NKT-like cells in individual subjects. (b) Summarized data show the number of CD158a^+^ NK cells in COPD patients and HNS. The horizontal lines show the median. (c) Summarized data show the number of CD158b^+^ NK cells in COPD patients and HNS. (d) Summarized data show the number of CD158b^+^ NKT-like cells in COPD patients and HNS.

As mentioned above, NK and NKT-like cell functions are associated with a balance of activating and inhibitory receptors [16]. Therefore, we analyzed the expression of NK and NKT-like cell receptors including NKG2C, NKG2D, NKp30, NKp46, CD158a, CD158b, KIR3DL1, and NKG2A. We found that the numbers of CD158a^+^ and CD158b^+^ NK cells as well as CD158b^+^ NKT-like cells were significantly greater in COPD patients compared with HNS (*P* = 0.003, *P* < 0.001, *P* = 0.007, resp.) (Figure 3). Differences between the other receptors were not significantly different between COPD patients and HNS (data not shown).

### 3.4. The Frequency of CD158b^+^ NK Cells Was Negatively Correlated with Pulmonary Function in COPD Patients

**Figure 4:**
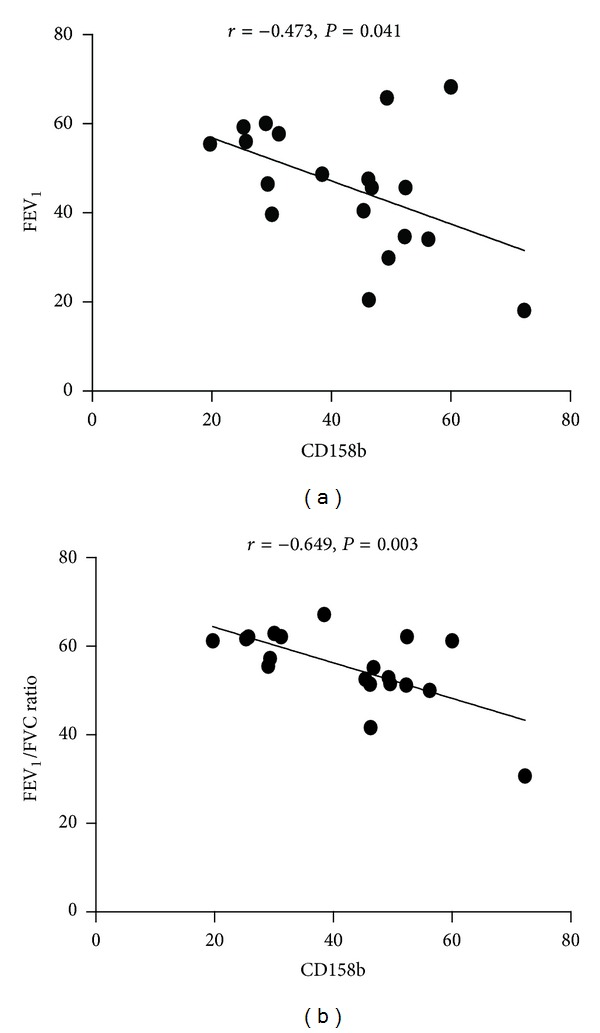
The correlation between FEV_1_, FEV_1_/FVC ratio and the frequency of CD158b^+^ NK cells in COPD patients. (a) The correlation between FEV_1_ and the frequency of CD158b^+^ NK cells. (b) The correlation between the FEV_1_/FVC ratio and the frequency of CD158b^+^ NK cells.

Pulmonary function, especially FEV1_1_% prediction and the FEV_1_/FVC ratio, is often used to evaluate the severity of COPD. For this reason, we analyzed the correlation between pulmonary function and lymphocyte subsets. We found that there was no correlation between pulmonary function and the frequencies of CD3^+^ T cells, CD4^+^ T cells, CD8^+^ T cells, NK cells, CD158a^+^ NK cells, CD158b^+^ NKT-like cells, and B cells in COPD patients (data not shown). However, the frequency of CD158b^+^ NK cells was negatively correlated with the FEV_1_% prediction and the FEV_1_/FVC ratio (*r* = −0.473, *P* = 0.041; *r* = −0.649, *P* = 0.003, resp.) (Figure 4).

## 4. Discussion

The results of the present study showed that the numbers of CD3^+^ T cells and CD4^+^ T cells and the CD4^+^/CD8^+^ ratio were significantly less in COPD patients compared with HNSs. These findings are similar to those reported previously [3–5]. However, the number of CD8^+^ T cells found in this study is different from other studies [7]. T cells can cause tissue injury either by direct cytolytic activities or through the secretion of proinflammatory mediators that recruit and activate other immune cell types [25]. CD4^+^ T cells are critical drivers of the B cell-dependent autoantibody response [26] and act as regulatory elements in the activation as well as the deletion of CD8^+^ T cells [5]. CD8^+^ T cells play a destructive role in COPD and can release proteolytic enzymes such as granzyme, which causes the death of structural cells [15, 27]. A decreased CD4^+^/CD8^+^ ratio is a characteristic feature of the pulmonary inflammatory response in COPD [27, 28], which is consistent with our results. Our data indicate that there is an imbalance of immune function in COPD patients, and this may explain why COPD patients are more prone to infections. B cells are the most important effector cells of humoral immunity. Active B cells recognize the antigen, develop into plasma cells that secrete immunoglobulins, and are finally converted to memory cells. Active T cells secrete cytokines, such as IL-2, IL-4, and IL-5, and act on B lymphocytes to induce proliferation [26]. In the present study, the number of B cells was not significantly different between COPD patients and HNS. Moreover, no correlation between B cells and pulmonary function was found.

NK cells are the first line of defense against infection in the innate immune system because they can be activated by cytokines [29]. When activated, NK cells will directly kill target cells through the release of cytolytic granules by forming pores in the membrane of target cells [30]. NKT cells exhibit features of both T cells and NK cells and contribute to combating intracellular pathogens [13]. Our data revealed that the numbers of CD3^−^CD56^+^ NK cells and CD3^+^CD56^+^ NKT-like cells were significantly greater in COPD patients compared to HNS, indicating that CD3^−^CD56^+^ NK cells and CD3^+^CD56^+^ NKT-like cells may be involved in the pathogenesis of COPD. However, these results are not consistent with previous findings [14, 15]. In homeostasis, the function of NK cells in bronchoalveolar lavage fluid or lung tissue is suppressed; although they can conjugate with target cells, their cytotoxic capacity is profoundly impaired [31, 32]. Once infection has developed, large numbers of NK cells are recruited into the lung, and then they are activated to secrete cytokines, mainly IFN-*γ* [33]. Subsequently, we studied the functions of NK cells and NKT-like cells by detecting the secretion of IFN-*γ* and CD107a degranulation in these cells. We found that the frequencies of IFN-*γ*-secreting NK cells and NKT-like cells were significantly less in COPD patients than in HNS. These data indicate that NK and NKT-like cell functions are impaired in COPD patients, which explains why COPD patients are more susceptible to infection.

NK and CD3^+^CD56^+^ NKT-like cell functions are associated with a balance of activating and inhibitory receptors. Our data indicate that NK and CD3^+^CD56^+^ NKT-like cell functions are impaired in COPD patients; therefore, we analyzed the activating and inhibitory receptors of NK cells and CD3^+^CD56^+^ NKT-like cells. Finally, we showed conclusively that the inhibitory receptors CD158a and CD158b on NK cells and CD158b on NKT-like cells were significantly greater in COPD patients compared with HNS. No differences in the other receptors evaluated were observed. CD158b is a killer cell immunoglobulin-like receptor (KIR) that has specificity for HLA-C antigen [34]. CD158b contains D1 and D2 extracellular immunoglobulin-like domains and a long cytoplasmic tail containing immunoreceptor tyrosine-based inhibition motifs (ITIMs) that recruit and activate SHP-1 and SHP-2 phosphatases for inhibitory signal transduction [35, 36]. CD158a, also an inhibitory receptor, is similar in structure and function with CD158b. Accordingly, we presume that impaired NK and CD3^+^CD56^+^ NKT-like cell functions may be associated with increased inhibitory receptors CD158a and CD158b.

The dysfunction of lymphocyte subsets was observed in COPD patients. To demonstrate whether this dysfunction correlated with the severity of COPD, we assessed the correlation between the frequency of lymphocyte subsets and pulmonary function. Different from other studies showing that COPD severity is significantly and inversely associated with the frequency of circulating CD4^+^ T cells and the CD4^+^/CD8^+^ ratio [37], no such correlation was observed in our research. This difference may be due to the patients' respiratory conditions and matching the condition to pulmonary function determination. Through further correlation analysis between NK cell subsets and pulmonary function, we found that the frequency of CD158b^+^ NK cells was negatively correlated with FEV_1_ and the FEV_1_/FVC ratio. No correlation was observed between the frequencies of CD158a^+^ NK cells or CD158b^+^ NKT-like cells and pulmonary function. Thus, our data indicate that the frequency of CD158b^+^ NK cells reflects the severity of COPD to a certain extent.

## 5. Conclusion

In conclusion, our data indicate that there is an imbalance of immune function in COPD patients. Increased numbers of NK cells and NKT-like cells expressing inhibitory receptors may participate in the pathogenesis of COPD, and the frequency of CD158b^+^ NK cells may reflect the severity of COPD to a certain extent.

## Figures and Tables

**Table 1:** Clinical and laboratory characteristics of the COPD patients and HNS.

Characteristic	HNS (*n* = 12)	COPD (*n* = 19)
Male/female	6/6	10/9
Age (years)	54.75 ± 6.47	55.84 ± 5.72
Onset time (years)	0	20 ± 7.41
WBC count (×10^9^/L)	5.22 ± 1.02	6.04 ± 1.76
FEV_1_ (%)	88.28 ± 6.13	46.02 ± 14.20*
FEV_1_/FVC ratio	76.69 ± 2.69	55.3 ± 8.60*
Hyperinflation/enhancement of pulmonary markings	0	19

Data are shown as average ± SD; WBC: white blood cell count, FEV_1_: forced expiratory volume in one second, FVC: forced vital capacity, *P < 0.05 versus the HNS.
